# Coordination between binocular field and spontaneous self-motion specifies the efficiency of planarians’ photo-response orientation behavior

**DOI:** 10.1038/s42003-018-0151-2

**Published:** 2018-09-21

**Authors:** Yoshitaro Akiyama, Kiyokazu Agata, Takeshi Inoue

**Affiliations:** 1 Department of Biophysics, Graduate School of Science, Kyoto University, Kitashirakawa-Oiwake, Sakyo-ku, Kyoto, 606-8502 Japan; 2 Department of Advanced Interdisciplinary Studies, Graduate School of Engineering, The University of Tokyo, 4-6-1 Komaba, Meguro-ku, Tokyo, 153-8904 Japan; 3 Department of Life Science, Faculty of Science, Gakushuin University, 1-5-1 Mejiro, Toshima-ku, Tokyo, 171-8588 Japan

## Abstract

Eyes show remarkable diversity in morphology among creatures. However, little is known about how morphological traits of eyes affect behaviors. Here, we investigate the mechanisms responsible for the establishment of efficient photo-response orientation behavior using the planarian *Dugesia japonica* as a model. Our behavioral assays reveal the functional angle of the visual field and show that the binocular field formed by paired eyes in *D*. *japonica* has an impact on the accurate recognition of the direction of a light source. Furthermore, we find that the binocular field in coordination with spontaneous wigwag self-motion of the head specifies the efficiency of photo-responsive evasive behavior in planarians. Our findings suggest that the linkage between the architecture of the sensory organs and spontaneous self-motion is a platform that serves for efficient and adaptive outcomes of planarian and potentially other animal behaviors.

## Abstract (web summary)

Yoshitaro Akiyama et al. report the use of innovative behavioral assays in planarian flatworms to investigate the mechanism by which they efficiently respond to light. They find that binocular vision and spontaneous self-motion are key factors for accurately detecting the direction of a light source.

## Introduction

Behavior based on visual cues is common in the animal kingdom and is achieved by a process that comprises receiving optical signals through visual neurons, integrating them in the brain, and making a decision about the appropriate response. The morphology of eyes is diverse among creatures in different ecological niches and during evolution, suggesting that the morphological traits of eyes adapt to match animals’ visual behaviors^1,2^. In particular, the photo-response orientation behavior of simple animals is one of the strongest ecological factors directly and indirectly contributing to biomass migration^3,4^ and reproduction^5^. However, the mechanisms responsible for the accuracy of photo-perception, those underlying the architectural features of the eyes, and the internal processing mechanisms contributing to efficient directional movements remain largely unknown although they are highly relevant to many questions regarding the physiology and evolution of visual properties.

The planarian *Dugesia japonica* (phylum Platyhelminthes) belongs to an evolutionarily basal group of animals possessing a pair of simple eyes and a brain^6,7^, and it exhibits robust evasive behavior in response to light (known as negative phototaxis)^8–10^. Planarian eyes, which share genetic similarities with vertebrate eyes^11–14^, are composed of two cell types: pigment cells that are arranged into a semilunar eyecup, and visual bipolar neurons that consist of cell bodies, rhabdomeres^15^, and axons^16^. These axons form a hemidecussation^17^ and project directly to the brain^18^. It was suggested that although the planarian eye is nondioptric and cannot recognize images^9^, the visual neurons respond to light from only one side due to shading by a pigment cup^19^.

The planarian brain consists of several structural domains that are defined by a complex set of genes and neural networks^11,18,20–22^. The combination of behavioral assays quantifying many parameters and RNAi to knockdown neuron-specific genes has demonstrated that neural networks in planarians strictly regulate distinct behaviors via the corresponding sensory organs and brain neurons in response to specific environmental stimuli^23–27^. For example, using region-specific RNAi (*Readyknock*) of a neuron-specific gene, *snap25* (synaptosome-associated protein of 25 kDa), it was shown that planarians receive light signals from visual neurons, and that these signals may be processed in the brain^24^. It was also reported that GABAergic neurons in the brain might be involved in the information-processing of the light signals transmitted from visual neurons, as revealed by RNAi experiments to knockdown the *gad* gene, which encodes a rate-limiting enzyme for the synthesis of GABA^28^. In addition to behaviors performed in response to a single environmental stimulus, planarians exhibit decision-making behaviors in response to simultaneously provided environmental stimuli, indicating that planarians decide upon behavioral strategies by integrating multiple external signals in their brain. However, the mechanisms underlying robust photo-response orientation behavior have not yet been elucidated.

More recent work showed that planarians sway their heads horizontally, and this swaying motion (called the wigwag self-motion) is sustained even after removal of the head from the rest of the body, in the absence of any environmental cues or spatial information^29^, indicating that this motion occurs spontaneously independent of brain activity. Interestingly, although planarians have a behavioral trait of proceeding along a wall, the angle and the frequency of the wigwag self-motion determine the probability of staying near the wall or leaving it even in the absence of any environmental cues and are optimized for sustaining the proper distance from the wall, suggesting that the brain-independent spontaneous self-motion in planarians plays crucial roles in some adaptive behaviors, such as hiding in a concave space. However, the relationship between spontaneous self-motion and environmental stimulus-associated directional movement behavior in planarians has not yet been investigated.

Therefore, planarians, with their simple eyes, brain, robust behavioral properties, and evolutionary position, provide unique models for investigating the pivotal functions of the perception of the light direction in bilaterians^30^. Here, using rigorous photo-response orientation behavior assays with the planarian *D. japonica* we obtained insight into how the architecture of the simple eyes enables precise photodetection and demonstrated that *D*. *japonica* possesses an anterior binocular field, which in association with spontaneous self-motion contributes to establishing efficient and adaptive behavioral outcomes.

## Results

### *D. japonica* has an anterior binocular field

**Fig. 1:**
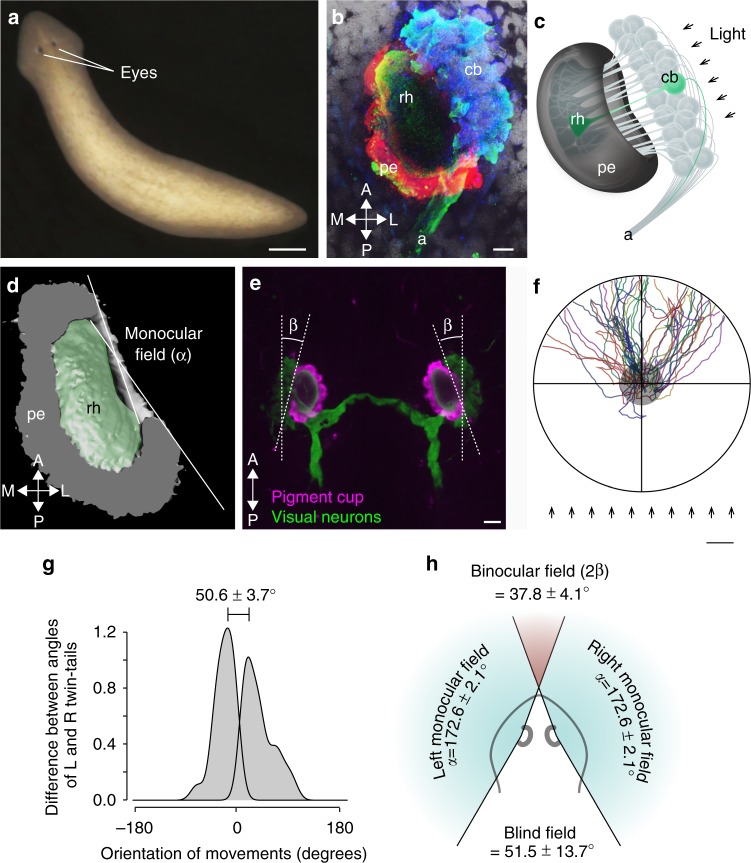
Eye morphology and photo-response orientation behavior of the planarian *D. japonica*. **a** Live *D. japonica*. Scale bar, 500 µm. **b** Magnified view of a right eye in *D. japonica*. Blue, cell bodies of visual neurons (cb) visualized by in situ hybridization with *opsin* gene probe; green, axons and rhabdomeres (rh) visualized by immunostaining using anti-arrestin antibody; red, pigment eyecup cells (pe) visualized by immunostaining using anti-TPH antibody; white, nuclei visualized by staining with Hoechst. A, anterior; P, posterior; M, medial; L, lateral. Scale bar, 10 µm. **c** Schematic drawing of architecture of planarian eye indicated in **b**. **d** Ventral semiellipsoid of a 3D reconstruction of the right eye based on confocal microscopic sections with the indication of a monocular field. *D. japonica* has a visual field of 172.6 ± 2.1° in one eye. *n* = 6. **e** The eyes of *D. japonica* are oblique (with an angle ≈ *β*). The angle of the obliqueness is approximately 20° (*β* = 19.4 ± 2.0°) on average. *n* = 28. Scale bar, 25 µm. **f** Distribution of traced trajectories of movements in the orientation assay with one light source (OA1L). Each colored line indicates the trajectory of an individual. The center of the assay field indicated by a gray circle shows the start area. Arrows indicate rays of light. Although animals show evasive movement away from the light source, a plot of this movement for a large number of animals shows a wide fan-shape composite plot. The 300 lux in the assay field was used. Scale bar, 1 cm. **g** Distribution of the difference between angles of trajectories toward the left and toward the right shown by a density plot. The difference was 50.6 ± 3.7° wide. *n* = 41. **h** Schematic drawing of planarian visual field. Binocular field on the anterior side of planarian *Dugesia japonica* is approximately 40° (2*β* = 37.4 ± 4.1°), Blind field on the posterior side is approximately 50°, and the planarian visual field in one eye is greater than 170°

When we stained the cell bodies of visual neurons, the dendrites and axons of visual neurons, and pigment cells of planarian eyes (Fig. 1a) using specific markers^6,14,16,31^, the structure of the planarian visual system was visualized (Fig. 1b, c). The monocular visual field (*α*) of a planarian eye was greater than 170° (172.6 ± 2.1°), and was slightly tilted anteriorly relative to the body’s lengthwise axis in the horizontal plane, as revealed by three-dimensional reconstructions of the pigment cup and rhabdomere outline (Fig. 1d). The eyes are oriented obliquely relative to the anterior–posterior axis at an angle (*β*), which provides a binocular field (≈2*β*) of approximately 40° (37.8 ± 4.1°, mean ± standard deviation (SD), *n* = 28) on the anterior side (Fig. 1e).

In order to investigate the accuracy of the recognition of the light direction by *D. japonica*, we developed a photo-response orientation assay specialized for analyzing the orientation of movement relative to the incident light, named the orientation assay with one light source (OA1L), and examined the trajectories of the movement of illuminated animals. Animals strongly avoided light (Fig. 1f). However, OA1L revealed that animals did not move straight away from the light source; trajectories fell into two clusters that were twin-tailed. The difference between the angles of the escape trajectories toward the left versus toward the right of *D. japonica* was 50.6 ± 3.7° (Fig. 1g, Supplementary Fig. 1), which was consistent with the geometric arrangement of the blind field (51.5 ± 13.7° = 2(180 − *α* + *β*)) on the posterior side (Fig. 1h), indicating that the direction of movement away from the light source strongly correlated with the eye architecture in planarians. These data indicate that this novel assay is sufficiently sensitive to enable measurements of the angle of the visual field, and also that the morphological features of the eyes affect photo-response orientation behavior in planarians.

### Planarians evaluate the difference of the eyes’ input

**Fig. 2:**
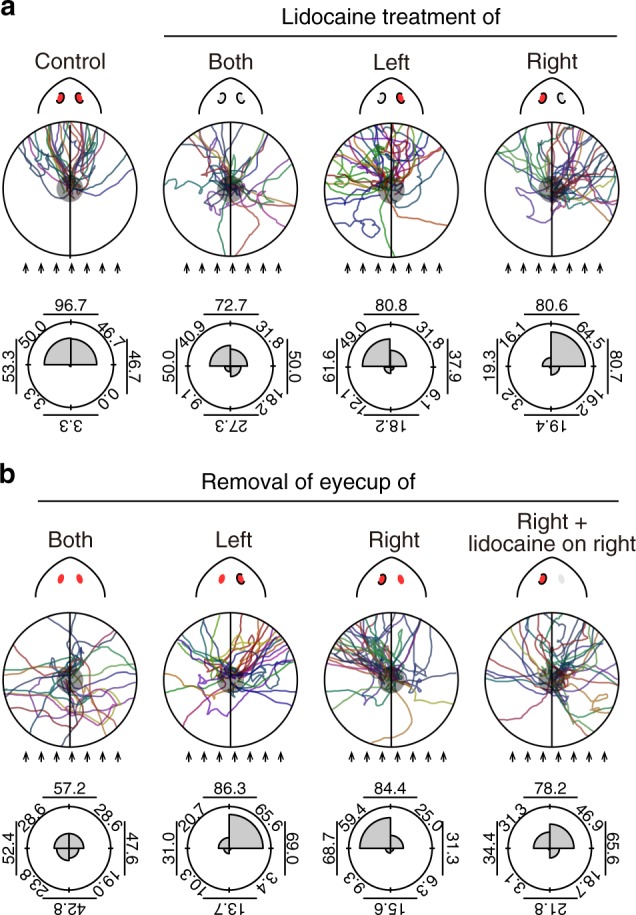
Planarian recognizes the light direction by comparing the difference of the input from the two eyes. **a** Trajectories of lidocaine-treated planarians. The control group was treated with anesthesia just below the right eye. The trajectories of the group administered lidocaine to both eyes were perturbed (Both), indicating that the lidocaine treatment efficiently inhibits the activity of visual neurons. Trajectories of the group administered lidocaine to the left eye was biased toward the left (Left), while that of the group administered lidocaine to the right eye was biased toward the right (Right). Rose plots in the lower panels show the histogram of the orientation (angle) distribution of movement of lidocaine-treated planarians, determined as the average for individuals used for this assay. The angle of the direction of each individual was calculated and the data were binned into 90° intervals. The percentage of oriented movements in the range of four every 90° interval, half angle against the light source, half angle toward the light source, the half angle on the right side, and half angle on the left side are shown in each plot. **b** Trajectories of eyecup-removed planarians. The trajectories of the group with the removal of both eyecups (Both) were strongly perturbed and more random than those of the control. The trajectory of the left eyecup-removed group was significantly biased in the right direction (Left), while that of the right eyecup-removed group was significantly biased in the left direction (Right). Furthermore, this biased behavior was rescued when lidocaine anesthesia was applied to the right eye (Right + lidocaine), indicating that the operated visual neurons functioned properly. Rose plots in the lower panels show the histogram of the orientation (angle) distribution of movement of eyecup-removed planarians, determined as the average for individuals used for this assay. *p* Values were less than 0.005 in the lidocaine-treated and eyecup-removed planarian groups, but not in the right eyecup-removed and lidocaine-treated group, relative to the control. Arrows indicate rays of light. *n* = 22–32

To investigate the interaction of optical signals received by the left and right eyes, we inhibited the activity of the visual neurons using an inhibitor of ionic influx (lidocaine) to alter the perception of light intensity between the two eyes. Control planarians administered lidocaine at a position adjacent to the visual neurons showed normal photo-response orientation behavior, whereas planarians administered lidocaine on both eyes showed dramatically perturbed behavior, with random directional trajectories (Fig. 2a). On the other hand, the trajectories of planarians lidocaine-treated on the left eye were biased toward the left, and vice-versa.

In addition, we removed the pigment eyecup (which normally blocks light coming from the corresponding side) of one eye. This eyecup removal increased the light input to the corresponding eye, enabling us to investigate whether the planarian recognized the addition or subtraction of the intensity of light coming from left versus right. The photo-response orientation behavior of planarians with removal of both eyecups was dramatically perturbed: they showed random directional trajectories, although their visual neurons and locomotor activity were intact (Fig. 2b, Supplementary Fig. 2a, b). The trajectory of left-eyecup-removed planarians, in which the input signal intensity of the left eye would be strengthened, was significantly biased in the right direction, which was consistent with the behavior of planarians with lidocaine anesthesia of the right eye. Conversely, the trajectory of right-eyecup-removed planarians was significantly biased toward the left. When we treated the eyecup-less right eye with lidocaine to decrease the signal input, the orientation of movement reverted toward normal, with less-biased orientation toward the left.

Furthermore, *menashi* mutant planarians^32^, which naturally lack eyecups (although their visual neurons are intact), did not show normal photo-response orientation behavior (Supplementary Fig. 2c, d). This result is consistent with the behavior of *tryptophan hydroxylase* (*tph*)*(RNAi)* planarians, which lack melanin pigmentation in the eyecup^33^. The above data indicated that planarians recognized the light direction by assessing the laterality of the signal inputs received by the two eyes, and the input value of the light in each eye is approximated by the eye’s surface area receiving light in planarians, independent of the light intensity^34^ (Supplementary Fig. 2e).

### Binarized response via the GABAergic neural network

**Fig. 3:**
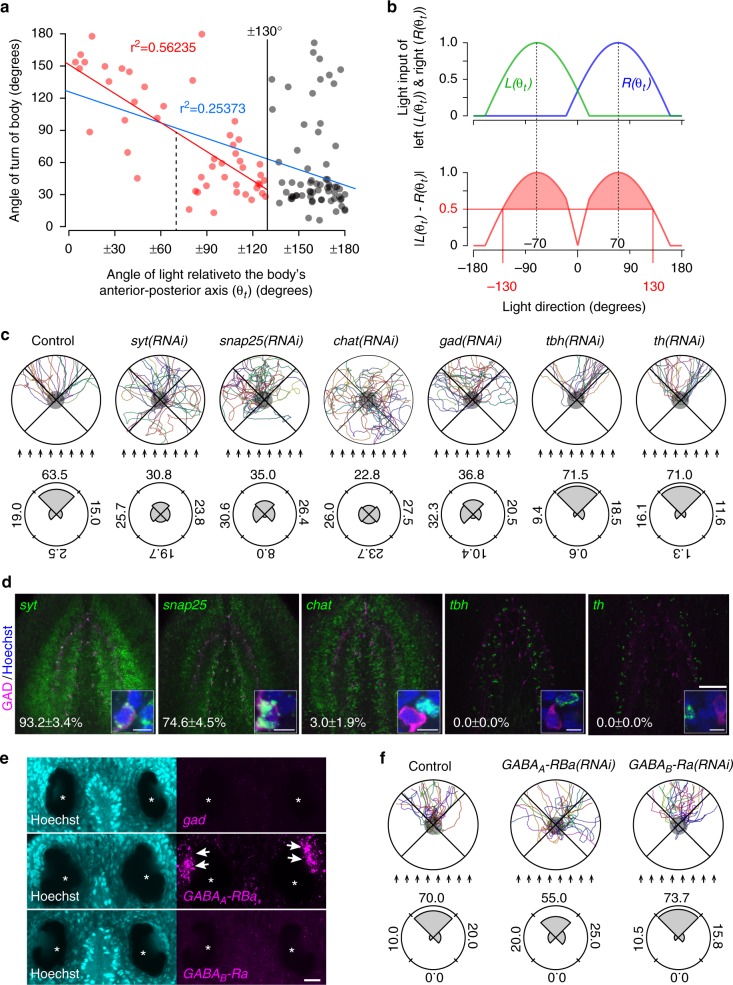
Integration of input signals in the brain for the induction of body responses. **a** Scatter plot of turn angles caused by incident light from different directions. 0° corresponds to anterior end, and ±180° corresponds to posterior end, respectively. All points are classified into two colors (black or red) at the dividing line of 130°. The coefficient of determination indicated by the blue solid line calculated from all dots (red and black) showed low correlation (*r*^2^ = 0.25). The coefficient of determination indicated by the red solid line calculated from the red dots showed a significant correlation (*r*^2^ = 0.56) that corresponded to a light angle of ±130° when the difference in the signal input value between the L and R eyes exceeded the response threshold to induce precise turning (vertical black solid line at 130°). **b** Input value to the left and right eyes (top) and subtractive difference in the left and right eyes (bottom) with a change in the light direction. The response threshold calculated using the subtractive formula |*L*(*θt*) − *R*(*θt*)| corresponding to the light angle of ±130° is 0.5 (red shaded) between the light input received by the two eyes. Maximum input is assumed to be 1.0. **c** The trajectories of control (GFP-dsRNA injected), *syt(RNAi)*, *chat(RNAi)*, *snap25(RNAi)*, *gad(RNAi)*, *tbh(RNAi)*, and *th(RNAi)* planarians and their histograms of the orientation (angle) distribution indicated by rose plots. Percentage of oriented movements in the range of four for every 90° interval on the rose plots. **d** Fluorescence immunohistochemistry of GAD proteins combined with fluorescence in situ hybridization of *syt*, *snap25*, *chat*, *tbh*, *th* genes. Percentages (mean ± SEM) of GAD-positive neurons co-expressing neurotransmitter-related genes are shown in the lower left corners. Scale bars, 50 µm. Inset scale bars, 5 µm. **e** Expression patterns of the planarian *gad*, *GABAA-RBa*, and *GABAB-Ra* genes in the eyes. Arrows indicate visual neurons expressing *GABAA-RBa*. Asterisks indicate pigment eyecups. Scale bar, 20 µm. **f** The trajectories of control (GFP-dsRNA injected), *GABAA-RBa(RNAi)*, and *GABAB-Ra(RNAi)* planarians and their histograms of the orientation (angle) distribution indicated by rose plots. Arrows indicate rays of light

To examine the coefficient of determination for the induction of body turning based on the value of the difference between left and right inputs, we measured the angle of a planarian’s turn away from the direction of the light (*θt*), and then performed regression analyses using data obtained at different angles of light. Plots of the turning angles of the body in response to light irradiated from various different angles indicated that planarian showed avoidance behavior to light received from an angle of less than 130° relative to the anterior–posterior body axis (red dots) (Fig. 3a). In contrast, when planarians received light from an angle of more than 130° (black dots), the turning angles were random. A regression analysis showed a correlation when using the turning angle with respect to a light angle of 130° or less (red line), whereas a greater light angle caused a weaker correlation (blue line). Since the input signal value of planarian eyes is approximated by the surface area of the eye receiving light, as represented by a sine function^10,19^, the light inputs of the left and right eyes are obtained as *L*(*θt*) and *R*(*θt*), respectively (Supplementary Fig. 3a, b). The minimum difference in the signal input value (response threshold) corresponding to the light angle of 130°, which is the borderline light angle for inducing a body movement reaction, was calculated by the subtractive formula |*L*(*θt*) - *R*(*θt*)| (Fig. 3b). The result obtained indicated that planarians are induced to turn their bodies away from the light source at a difference of more than 0.5 (maximum input assumed to be 1.0) between the light inputs received by the two eyes. Our results indicate that the variation of trajectories caused by the arrangement of the pigment eyecup shield inevitably creates a blind area and the response threshold (Figs. 1 and 3a, b). Therefore, to further evaluate the parameters of the response threshold, we performed another photo-response orientation behavior assay, named the “orientation assay with two light sources (OA2L)” to exclude a blind area in planarians. Trajectories showed bow-tie shaped composite plots (Supplementary Fig. 3c), which indicated that the animals escaped in paths perpendicular to both of the two light sources without a blind field on the posterior end, and supported the notion that planarian recognize the light direction by determining the input difference between the two eyes. Simulation analysis with different values of the response threshold showed that the response threshold of 0.5 resulted in trajectories that accorded with the actual trajectories, in contrast to those at a response threshold of 0.6 (Supplementary Fig. 3d, e). This result indicated that the response threshold affects the photo-response orientation behavior in planarians and the value estimated by performing OA2L is consistent with the results of the regression analysis (Fig. 3a, b).

When we tested OA1L with *D. japonica* possessing a supernumerary eye^16^, the planarians showed normal evasive behavior (Supplementary Fig. 4a, b), suggesting that the brain to which visual neurons project, not the eyes, may evaluate the disparity between the signal input and the response threshold. To identify the brain neural networks that define the response threshold for achieving light-evasion behavior, we performed OA1L using planarians treated with RNAi of neurotransmission-related genes (Fig. 3c). Although both the *synaptotagmin* (*syt*) and *snap25* genes are widely expressed in the brain (Fig. 3d), *syt(RNAi)* planarians and *snap25(RNAi)* planarians showed slightly different photo-response orientation behavior (Fig. 3c). The *syt(RNAi)* planarians could not recognize the direction of the light and showed random movements, whereas *snap25(RNAi)* planarians did not move toward the light source; instead, *snap25(RNAi)* caused broadened escape trajectories^24^ (Fig. 3c). To identify the neuronal subtype recognizing the laterality between the left and right inputs, we used RNAi planarians of genes encoding rate-limiting enzymes for neurotransmitter synthesis. RNAi planarians of *choline acetyltransferase* (*chat*) showed random trajectories, which were similar to the trajectories of *syt(RNAi)* planarians, whereas RNAi of the *tyramine β-hydroxylase* (*tbh*) and *tyrosine hydroxylase* (*th*) genes did not affect the photo-response orientation behavior. RNAi of *glutamic acid decarboxylase* (*gad*) caused broad trajectories, although *gad(RNAi)* planarians did not move toward the light source, a property that was the same as that of *snap25(RNAi)* planarians. When double staining analysis was performed, we found that GABAergic neurons co-express the *syt* and *snap25* genes, but do not co-express other genes encoding neurotransmitter-synthesis-limiting enzymes, including the *chat* gene (Fig. 3d). Moreover, the *syt* and *chat* genes are expressed in the visual neurons, but *snap25* and *gad* are not (Fig. 3e, Supplementary Fig. 4c). These results suggest that the *syt* and *chat* genes may be involved in transmitting the signals from visual neurons to the brain, and then GABAergic neurons co-expressing *snap25* may process the input signals received by the visual neurons. Further RNAi experiments to knockdown the *GABAA receptor* gene, which is expressed in visual neurons, or the *GABAB receptor* gene, which is not expressed in the visual neurons (Fig. 3e, Supplementary Fig. 4d, e), showed broad trajectories in the *GABAA-RBa(RNAi)* planarians, but not the *GABAB-Ra(RNAi)* planarians (Fig. 3f). Collectively, these results suggested that the GABAergic neural pathway between the eyes and the brain might amplify the difference between the left and right inputs by mutual inhibition of the signal received from the eyes (Supplementary Fig. 4f).

### Planarians do not recognize the illuminance gradient

**Fig. 4:**
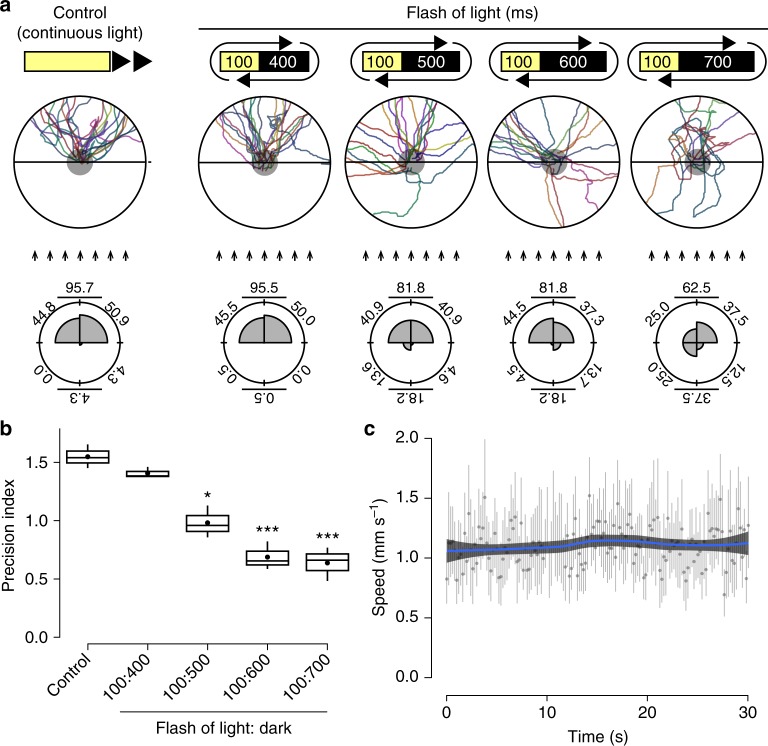
Time resolution of light exposure required for photo-response orientation behavior. **a** Distribution of traced trajectories of movements in OA1L using 100-ms flashes of light with several different intervals between flashes. Control, continuous light exposure from one direction. “100:400”: 100-ms light exposure and 400-ms dark interval; “100:500”: 100-ms light exposure and 500-ms dark interval; “100:600”: 100-ms light exposure and 600-ms dark interval; “100:700”: 100-ms light exposure and 700-ms dark interval. Arrows indicate rays of light. As the dark interval increased from 400 to 700 ms, the trajectories of movement became wider and more randomly oriented. *n* = 22. Rose plots in the bottom panels show the histogram of the orientation (angle) distribution in the range of 90° intervals of movement during the assay for several values of the interval between flashes. Percentage of oriented movements in the range of four per every 90° interval, half angle against the light source, and half angle toward the light source are shown on each plot. The movements of control planarians exposed continuously to light from a particular direction, and of planarians exposed to flashes of light with long dark intervals, showed no particular orientation. (**b**) Precision index of the orientation of movements. Precision indexes are expressed as the inverse of the circular standard deviation of the orientation of trajectories. *n* = 22. ^*^p < 0.05, ^***^p < 0.005. **c** Planarian speed during movement. Gray graphs show the mean speed and standard deviation of speed at each time point, and blue lines show the polynomial trend line. *D. japonica* recognizes the light direction within a period of less than 500 ms or within a distance of less than 500 µm

Our next question was whether planarians can recognize the illuminance gradient. We considered two possible mechanisms for recognition of the illuminance gradient. One was approximation of the spatial difference of light illuminance received during their movement. The other was approximation of the spatial difference of the light gradient received by the individual visual neurons along the anteroposterior axis. In order to investigate these possibilities, we performed OA1L with flashes of light. Animals escaped from light with a 100-ms light exposure (a 500-ms period with a 400-ms dark interval), whereas under conditions of a longer dark interval, the light-avoidance behavior of animals was strongly perturbed (Fig. 4a). The precision index (the inverse of the circular SD) showed that the precision of orientation significantly decreased at a dark interval of more than 500 ms (Fig. 4b). These results indicated that a single 100-ms flash of light is a long enough period for comparing the difference of left and right input, but is not long enough for driving the precise photo-response orientation behavior. Rather, *D. japonica* required ~500 ms to recognize the light direction, even if 400 ms of the period was in the dark.

In addition, the speed of planarian movement is approximately 1.0 mm s^−^^1^ (Fig. 4c). Therefore, if planarians recognize the spatial difference of light illuminance received during their movement, they need to sense differences in light intensity within a distance of less than 0.5 mm. At a distance of 0.5 mm, the difference in illuminance is less than 1.0 × 10^−10^ lux under natural sunlight, and even in our laboratory test conditions, the difference of the illuminance is 0.24 lux under the condition of illumination with 5000 lux of light from a distance of 300 mm. Regarding the visual neurons along the anteroposterior axis, the distance between the anterior visual neurons and posterior visual neural cells in an eye is less than 100 µm. Highly sensitive detection of tiny differences in light intensity over a short distance for the integration of the light input in the brain would thus be necessary for precise photo-recognition. These results suggest that planarians do not recognize the illuminance gradient in photo-response orientation behavior via either approximation of the spatial difference of light illuminance received during their movement or the difference of the light gradient received by the individual visual neurons along the anteroposterior axis, and therefore a mechanism different from the above two mechanisms should underlie the photo-response orientation behavior in planarians. Also, our results gave rise to a paradox regarding how planarians distinguish between the directions of light from precisely their anterior and posterior ends, because the inputs of both eyes are equal in these two cases.

### The binocular field affects the photo-response efficiency

**Fig. 5:**
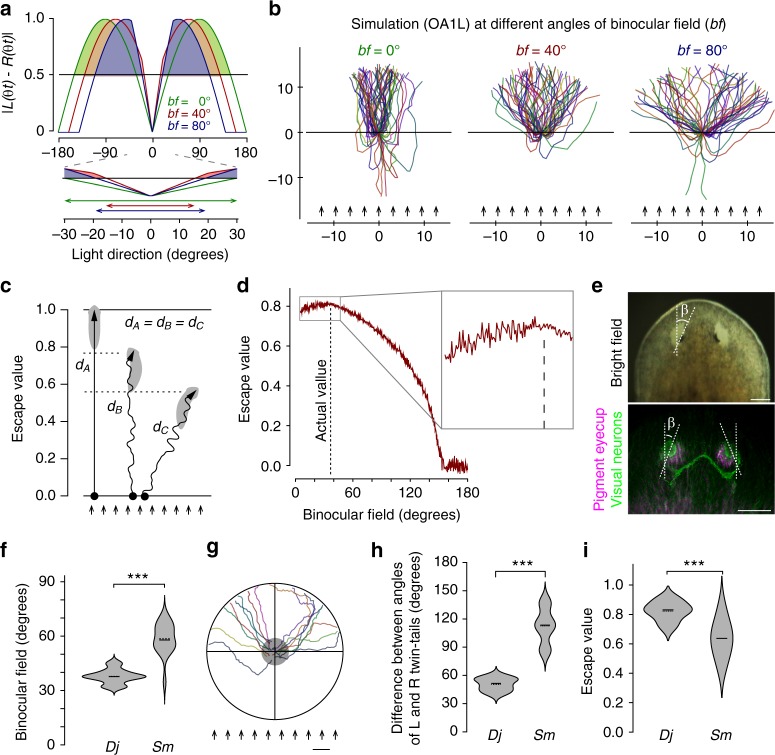
A binocular field of 40° is optimally tuned to achieve maximal efficiency of photo-response orientation behavior. **a** Subtractive difference of inputs from the two eyes with a change in the light direction and binocular field parameters (*bf*) of 0° (green), 40° (red), and 80° (blue) (upper panel). The value of the response threshold caused an angle that could not be distinguished from the light direction on the anterior side (front blind-like spot), and the angle of front blind-like spot differed depending on the angle of the binocular field. Filled colored areas of the graph indicate the angle greater than the response threshold. Lower panel shows higher magnification of the front blind-like spot. Double-headed arrows indicate the angle of the front blind-like spot for different angles of binocular field of 0°, 40°, and 80°. **b** Simulated trajectories of photo-response orientation behavior in planarians with *bf* of 0°, 40°, and 80°. *n* = 60. Arrows indicate rays of light. **c** The “escape value” was defined as the relative distance from the start line from which ideal planarians showed an escape value of 1, moving straight away from a light source. **d** The simulated escape value was plotted versus different angles of the binocular field. The actual angle of the binocular field of *D. japonica* is shown by a dashed line. *n* = 1000. (**e**) Head and eyes of *S. mediterranea* visualized by immunohistochemistry of TPH (magenta) and arrestin (green). Scale bars, 100 µm. **f** Violin plot showing the comparison of the angle of the binocular field between *D. japonica* and *S. mediterranea*. *n* = 21. **g** Actual trajectories of *S. mediterranea* in OA1L showing broader twin-tailed trajectories. Arrows indicate rays of light. *n* = 16. Scale bar: 1 cm. **h** Difference in the twin-tail angles of *D. japonica* and *S. mediterranea*. **i** Escape values of actual trajectories of *D. japonica* and *S. mediterranea*. Horizontal solid lines indicate median value, and horizontal dashed lines indicate average value in violin plots in **f**–**h**, and **i**, respectively. ^***^p < 0.005

In order to investigate the role of the binocular field in the robust detection of the direction of light in planarian photo-response orientation, we performed a simulation analysis with different angles of the binocular field, and the results obtained showed that the absence of a binocular field (*bf* = 0°) resulted in narrow trajectories, which we speculate would enable high escape efficiency, whereas a binocular field of 80° (*bf* = 80°) caused broad trajectories due to the wider blind field in the posterior side and also decreased the disparity between the left and right inputs (Fig. 5a, b). The results of this simulation also showed that some individuals without a binocular field might move toward light and take a longer time to accurately recognize the light’s direction (*bf* = 0°).

In order to obtain a clearer understanding of the role of the obliqueness of the eyes in photo-response orientation behavior, we defined the relative distance from the start point from which an “ideal planarian” moves away from light for an arbitrary distance as having an “escape value” of 1.0 (Fig. 5c). Comparison of the escape values for different angles of the binocular field showed that the highest score of the escape value with few fluctuations was close to the actual angle of the binocular field in planarians, and also demonstrated that eyes with a binocular field of less than 30° or more than 40° resulted in a decrease in the escape value together with large fluctuations due to the stochastic appearance of individuals moving toward the light source (Fig. 5b–d).

In order to confirm the role of the binocular field in photo-response orientation, we tested another species of planarian, *Schmidtea mediterranea*, which has a wider binocular field than *D. japonica* (Fig. 5e, f). The actual composite trajectories of *S. mediterranea* in OA1L were broader twin-tailed trajectories (Fig. 5g), and the difference in the angle between the left and right twin-tailed paths of *S. mediterranea* was markedly larger than that of *D. japonica* (Fig. 5h). The escape value of *S. mediterranea* was lower than that of *D. japonica* in OA1L (Fig. 5i). These results indicate that the angle of the binocular field in planarians is an important bio-architectural feature that enables precise recognition of the direction of incident light.

### Spontaneous wigwag self-motion breaks illumination symmetry

Planarians recognize asymmetry in inputs to the two eyes when they are irradiated with light from an angle of approximately 15°–130° or an angle of approximately −15° to −130° relative to the anterior–posterior axis, corresponding to a difference in the signal input value of 0.5 or more (Fig. 3a, b). In addition, the value of the response threshold implied the existence of angles of illumination for which the light direction cannot be distinguished on the anterior side (front blind-like spot), since in the front blind-like spot the inputs to the two eyes are equal. In addition, the angle of the front blind-like spot differed depending on the angle of the binocular field (Fig. 5a). However, a regression analysis between the turn angle and the angle of incident light from −15° to 15° indicated that planarians clearly escaped from the light (Fig. 3a); therefore, we subsequently investigated the mechanisms by which planarians recognize the direction of light irradiated from the anterior end (from an angle of approximately −15° to 15°).

**Fig. 6:**
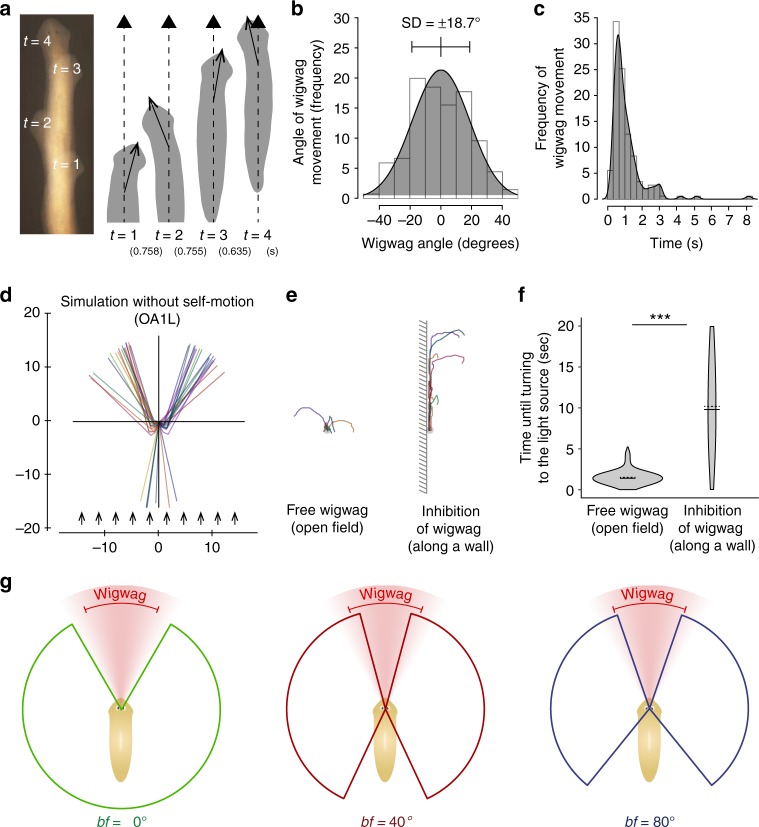
Wigwag spontaneous self-motion acts as symmetry breaking of the inputs from the two eyes. **a** Planarians show wigwag self-motion of the head even when they are moving straight ahead. Overlapped images of a time-lapse movie of movement (left). Traced drawings of wigwag self-motions of the head shown in the left panel (right). Numbers in parentheses in the bottom indicate the time between two wigwag self-motions. **b** The distribution of the wigwag angle fit follows a normal distribution with mean: 0, SD = 18.7°. *n* = 25 (143 turns). **c** The frequency of wigwag self-motions during movement follows a log-normal distribution with a log-mean of −0.15 s and log-standard deviation of 0.44. **d** Simulated trajectories without the wigwag parameter. *n* = 40. The simulation showed that some individuals go toward the light because the difference in the input signal between the two eyes may become as small when light comes from the anterior direction as when it comes from the posterior direction. Arrows, light source. **e** Turning assay using light irradiated from the anterior end in the open field and in the field partially obstructing wigwag self-motions along the wall. **f** Violin plot showing the time from the illumination by light source until movement in the opposite direction. Horizontal solid lines indicate median value, and horizontal dashed lines indicate average value in violin plots. *n* = 6–8. ^***^p < 0.005. **g** Schematic drawings of the relationship between the visual field and spontaneous self-motion. Colored sectors show the angles for which planarians can recognize the asymmetry between left and right inputs when the light comes from them (greater than the response threshold). Depending on the angle of the binocular field, planarians had a posterior blind field at different angles and a front blind-like spot at different angles, respectively. Sectors of red color gradation indicate the probability density distribution of the angles of wigwag motion, and red brackets indicate the standard deviation of the wigwag angle (±18.7°, total 37.4°)

Although planarians only glide forward using cilia^35^, in addition they constantly spontaneously sway (perform wigwag self-motion of) their head independent of brain function during movement^29^ (Fig. 6a). The distribution of the wigwag angle follows a normal distribution, and the SD of the change of head-angle during wigwag was ±18.7° (between inflection points indicated by SD) (Fig. 6b). The frequency of wigwag self-motions follows a log-normal distribution, and the average frequency of wigwag self-motions was once per 0.77 s (Fig. 6c). In order to investigate the role of wigwag self-motion in the photo-response orientation behavior, we performed the simulation analysis without the wigwag parameter. The result showed that most animals succeeded in escaping from the light source with composite twin-tail-shaped trajectories, with an angle of 58.1° between the twin-tailed paths (Fig. 6d), which was consistent with the actual observed trajectories (Fig. 1e, f). However, the simulation showed that some individuals moved toward the light (Fig. 6d). Consistent with this simulation result that planarians cannot distinguish between light from the anterior and posterior sides, we found that planarians occasionally turned their bodies by approximately 180°, even when they were exposed to light from the posterior side (±150 to ±180 relative to the body’s anteroposterior axis), although in almost all cases planarians make direction corrections of only small angles (black dots) (Fig. 3a). These results indicated that the mechanism employed by planarians to avoid going toward light does not depend solely on the binocular system; in addition, wigwag self-motion is required. When the angle of the front blind-like spot is greater than that of the wigwag self-motion, planarians move toward the light source until they sense the laterality of the light input as a result of the wigwag self-motion. These differences between the angle of the front blind-like spot and the angle of the wigwag self-motion are consistent with the observation that some individuals moved toward the light source in the absence of a binocular field (*bf* = 0°) or in the presence of a wide-angle binocular field (*bf* = 80°) (Fig. 5b). The restriction of the wigwag self-motion by a lateral wall caused planarians to move toward the light source for a longer time, whereas planarians in an open space promptly changed their orientation (Fig. 6e, f). This result indicates that this consistent escape behavior of planarians is due to the difference in the input signal between the two eyes becoming as small as the response threshold when light comes from an anterior direction (front blind-like spot: from an angle of approximately −15° to 15°) as when it comes from a posterior direction. In other words, planarians without wigwag self-motion cannot distinguish between light from the anterior and posterior sides. When the angle of the front blind-like spot is greater than that of wigwag self-motion, planarians lose the ability to detect the difference between the light inputs of the two eyes, and they may go toward the light source until they sense the laterality of the light input as a result of wigwag self-motion. The angle of the front blind-like spot (±15°, total 30°) of planarians possessing a binocular field of 40° is less than the value of the SD (±18.7°, total 37.4°) of the wigwag angle (Fig. 6g). In contrast, the angle of the front blind-like spot of planarians possessing either a 0° or 80° binocular field is greater than the value of SD of the wigwag angle. Thus, stochastically, 42% wigwag self-motion is larger than the front blind-like spot calculated using the standard normal distribution (see Methods). Since the frequency of wigwag motion is once every 0.7 s, planarians can turn away from the light within approximately 1.6 s even if planarians are facing the light source. In contrast, the probability that planarians possessing either a 0° or 80° binocular field would sway at larger angles than the front blind-like spot would be low, and consequently they would take more time to turn away from the light source.

Furthermore, we determined the optimal combination between the angles of the binocular field and wigwag angles for achieving efficient light-evasive behavior, and the results obtained were consistent with the actual values found in *D. japonica* (Supplementary Fig. 5a). Moreover, *S. mediterranea*, which possesses a wider binocular field, showed wider wigwag angles, indicating that the binocular field is correlated with spontaneous wigwag self-motion (Supplementary Fig. 5b, c).

## Discussion

Photo-response orientation behavior in planarians has been reported for more than a century^36^, and the working principles have been explained, but with some missing details. Here, we developed novel planarian photo-response orientation behavior assays that enabled us to examine the functional angle of the visual field, revealing the angle of the binocular field on the anterior side and a blind field on the posterior side in planarians. These assays revealed the mechanisms underlying the recognition of light directions, providing criteria for evaluating the precision of the recognition of light directions in planarians. We revealed the significance of the binocular field using a simulation analysis and comparative behavioral analyses with different angles of the binocular field, and revealed that simulated planarians that lacked a binocular field (*bf* = 0°) or that had a broad binocular field (*bf* = 80°) were predicted to have reduced precision of recognition of the light orientation. Our results show that the slight angle between the two eyes of planarians represented a functional trait for producing a binocular field that enables more precise recognition of the direction of incident light. It was previously stated that the diagonal trajectory of escape-behavior relative to a ray of light might be due to the eyes’ obliqueness decreasing the fidelity of the recognition of light directions in planarians^9,10^. In contrast, the results of this study indicate that the binocular field was acquired for establishing the robustness of the laterality of input signals between the eyes. Intriguingly, the eyes of multiocular planarian species, such as *Polycelis sapporo*, are also positioned at an angle relative to the anterior–posterior axis^37^, and vertebrates show interspecific variations in the angles of their eyes^38^, suggesting that the architectural basis for the recognition of light directions is conserved across diverse species of animals. A binocular field is involved in stereoscopic vision in vertebrates^39,40^ and in detecting moving targets in insects^41^, but planarians possess a novel function of the binocular field, namely, forming a blind-like spot in which planarians cannot distinguish the difference between left and right light inputs.

Although the architecture of the eyes is diverse among animals, animals as evolutionarily early as multicellular organisms without any nervous system^2,42,43^, as well as unicellular organisms^44^, had acquired screening pigments and a photosensory capacity in a single cell, indicating that the detection of the orientation of light direction by shielding against light coming from the opposite side represents an evolutionarily fundamental function of photosensing^34,45^. Our results suggested that changing of planarian’s body orientation away from a light source that is induced when the difference in the ratio of the signal input value between the eyes is greater than the value of the response threshold might be determined by the inhibitory GABAergic neural pathway in the brain. The formation of hemidecussation^17^ of the visual axons in planarians might also be the fundamental neural structure, which provides the capability to compare the light inputs between the two eyes by mutual inhibition via the GABAergic inhibitory neural pathway, as previously suggested^34^. The integration of inputs from the two eyes in the brain may not only improve behavioral efficiency by allowing more modifications, such as decision-making^46^, but may also bridge the gap between simple photo-response orientation behavior and more complex behavior, including animal navigation that requires cross interactions with other environmental cues, such as odor molecules and magnetic fields^46,47^.

In the chemical source localization of cells and insects, random motility increases the precision of sensing a chemical source^48,49^. Also, many planktonic organisms display spiral, conical, or horizontal motion during phototaxis^50–53^, suggesting that intrinsic self-motions are a basis for the precise directional movement in the animals. The spontaneous self-motion of the planarian head, the wigwag self-motion, which was previously considered to disturb responsive behaviors, was shown here to be necessary for ensuring the binocular field-based accurate perception of the light direction. These facts support the notion that intrinsic noise-driven excitability is generally employed for a number of sensory systems. Indeed, the suppression of the wigwag self-motion sufficiently explains some previous uninterpretable phenomena, i.e., that individuals occasionally did not respond to light or headed towards light^10,54,55^. In *S. mediterranea*, the wigwag angle is greater than that in *D. japonica*, and our data suggested that simulated planarians without a binocular field (*bf* = 0°) could efficiently escape if they had a much wider angle of wigwag self-motions that was greater than the broad front blind-like spot. However, it was reported that planarians moving with wider wigwag angles may lose the ability to stay in concave spaces such as on a stone or fallen leaves in the natural environment, although this ability is critical for avoiding toxic sunlight and strong water flow, when planarians cannot sense the environmental cues during head regeneration^29^. Thus, the wigwag self-motion and binocular field are strongly correlated and their angles presumably evolved in order to achieve multiple adaptive behaviors.

Planarians basically move straight ahead in the absence of any environmental stimulus^29^, indicating that planarians keep moving straight when the difference of left and right light input is less than the response threshold. Therefore, accurate light direction-perception based on the linkage between spontaneous self-motion and the eye architecture is simply implemented by the two motional characteristics: turning movement at over the response threshold of left and right input difference and straight movement at below the response threshold of left and right input difference. Previously it was observed that planarians responded to a weak light, but not to narrow bands of the spectrum, indicating that photo-response orientation behavior in planarian may not employ mechanisms detecting the illuminance gradient based on the difference of photoreactive chemicals in the nervous system, although we have not ruled out the possibility that planarians detect the light intensity for other behaviors. Moreover, temporal comparison between the light intensities at a certain position at time *t*_*n*_ and a previous position at time *t*_*n*−1_ requires saving this information in a memory system for illuminance or signal intensities at each time point, but planarian does not need this processing. We propose that planarian’s spontaneous self-motion that generates behavioral noise and its sensory architecture are co-adapted to enable the efficient, robust, and adaptive outcomes of multiple behaviors with potential reduction of energy consumption in the nervous system.

## Methods

### Animals

Three species of planarians: a clonal strain of freshwater planarian (SSP strain of *D. japonica*^20^, *Dugesia ryukyuensis* (*menashi* mutant strain)^32^ and *S. mediterranea*^56^) were used in the present study. They were cultured at 23°C in freshwater. Planarians that were 8 mm in length were used in all experiments. In the lidocaine treatment, animals were placed on two pieces of filter paper on ice to paralyze them, and 5 mg ml^−1^ lidocaine hydrochloride (Sigma) in 1.5% agarose gel (Takara) was then administered using a sharpened glass capillary under a microscope to block the activity of the visual neurons. The control group was treated with anesthesia just posterior to the eyes. Regarding eyecup removal, after animals were paralyzed on ice, the pigment eyecup was scraped out with a sharpened tungsten needle under a microscope. Behavior assays were performed 1 day after surgery, when visual neurons had healed and the pigment cup had not yet regenerated (Supplementary Fig. 2a). Control animals were scraped at a position next to the right pigment eyecup. All planarians were maintained and manipulated according to a protocol approved by the Animal Care and Use Committee of Kyoto University and Gakushuin University.

### Photo-response orientation behavioral assays

All behavioral experiments were conducted in a dark room with only a red light, the wavelength of which was not sensed by planarians^57,58^. The lid of a 9-cm plastic circular dish was placed on black paper to suppress reflection, and the central 8-cm portion of the dish (the arena) was used for the assay field. In OA1L, a light source with a condenser was set 30 cm away from the center of the assay field (yielding approximately 300 lux in the assay field). In OA2L, an additional light source of the same type was also set on the side of the assay field opposite the light source used for OA1L to remove the blind area (which inevitably affected variations in the orientation of movements due to the arrangement of the pigment eyecup shield). In light-flash experiments, a light source was connected to an electric digital stimulator (Nihon Kohden SEN-8203). The light intensity in the assay field was measured using an illuminance meter (Topcon, IM-5). To confirm the uniform intensity of illuminance of the assay field, pictures of the field were evaluated using ImageJ (National Institutes of Health). Planarians were kept in the dark for at least 60 min before the experiment, and then placed in random orientation in the center of the arena. Planarian behavior was recorded using a video camera (Sony HDR-CX700 or ILCE-7S) fixed above the assay field. Photo-response orientation behavior was recorded until animals crossed the edge of the arena (4 cm from the center), and tracking data were analyzed using a computer, SMART v2.0 behavior analysis software (Panlab), ImageJ, and R software.

### Statistical analysis

Data plotting and statistical analyses were performed using R software. The precision index of orientation was expressed as the inverse of the circular SD of average angles during movement using Eq. (1):

1 $${\mathrm{Precision}}\,{\mathrm{index}} = \frac{1}{{\sqrt { - 2\log R} }},$$

where *R* is the sample mean resultant length. The probability of wigwag angle larger than the front blind-like spot was calculated using the standard normal distribution Eq. (2):

2 $$F\left( x \right) = 2 \times \left( {{\int}_z^\infty {\frac{1}{{2\pi }}{\mathrm{exp}}\left( {\frac{{x^2}}{2}} \right)dx} } \right),$$

where *Z* was the angle of front blind-like spot.

In order to analyze differences between the angles of the left and right twin-tails, data on body angles were sorted into two groups: less than 180° as the left group, and greater than 180° as the right group, and the difference in the median value between the two groups was calculated. In order to measure body angle changes, we converted video files to image sequences. Images that showed the previous orientation (angle) relative to the orientation at the time point of a subsequent turning behavior were selected, head angles were measured using ImageJ, and body angle changes were calculated using these measurements. Data from at least three experiments were averaged to calculate the orientation (angle) of movement. Precision indexes are expressed as the inverse of the circular SD of the orientation of trajectories. Angles of the binocular field in planarians were calculated using images of gliding animals and fixed samples. Rao’s test for homogeneity was performed to assess differences in the dispersion of rose plot data. Watson’s two-sample test was performed to assess the significance of differences in the precision index. Regarding other data, the significance of differences between test results was evaluated using Student’s *t* test, Wilcoxon signed-rank test, Dunnett’s test, or the Tukey–Kramer test. The distributions of wigwag angles and frequency were fit by the maximum likelihood estimation method with a normal distribution and log-normal distribution, respectively. In all statistical tests, *p* values greater than 0.05 were considered to be not significant (ns).

### Computer simulation of planarian photo-response orientation behavior

All algorithms were implemented in Racket language. Output data were plotted using the Plot package. In order to generate a random number with a probability density that followed a normal distribution, Racket library math/distributions were used. The input signal was approximated with a sine function since the planarian pigment eyecup is semicircular (Fig. 1b, c, Supplementary Fig. 3). The functions for input signal intensities to the two eyes were expressed using Eqs. (3) and (4),

3 $$L\left( {\theta _t} \right) = - \sin \left( {\theta _t - \frac{{bf}}{2}} \right)$$

4 $$R\left( {\theta _t} \right) = \sin \left( {\theta _t + \frac{{bf}}{2}} \right)$$

respectively, where *θ*_*t*_ is the direction of light compared to the planarian anterior–posterior body axis at time *t* and *bf* is a binocular field parameter. The range of *θ* was limited to avoid the input signal intensity being a negative value. *bf* was assigned as 40 in the case of *D. japonica* or 60 in the case of *S. mediterranea*. If the difference in input signals was unacceptably large (over the response threshold (*τ*)), planarians changed their body angles in proportion to the difference, formulated using Eqs. (5) and (6),

5 $$\begin{array}{ccccc}\\ \varphi _{t \,+\, k} = & \varphi _t - 3\left( {\left( {R\left( {\theta _t} \right) - L\left( {\theta _t} \right)} \right) - {\mathrm{\tau }}} \right),\\ \\ & \left( {\left| {R\left( {\theta _t} \right) - L\left( {\theta _t} \right)} \right| > {\mathrm{\tau }} \wedge R\left( {\theta _t} \right) < L\left( {\theta _t} \right)} \right)\\ \end{array}$$

6 $$\begin{array}{ccccc}\\ \varphi _{t \,+\, k} = & \varphi _t + 3\left( {\left( {R\left( {\theta _t} \right) - L\left( {\theta _t} \right)} \right) - {\mathrm{\tau }}} \right),\\ \\ & \left( {\left| {R\left( {\theta _t} \right) - L\left( {\theta _t} \right)} \right| \,> \, {\mathrm{\tau }} \wedge R\left( {\theta _t} \right) \,> \, L\left( {\theta _t} \right)} \right)\\ \end{array}$$

where *φ* was defined as the planarian direction of movement with *φ* = −*θ*. The wigwag frequency (*k*) of the simulation was generated by a random number generator with a probability density that followed a log-normal distribution. The log-mean (*E*(*x*)) and log-standard distribution (*V*(*x*)) of the actual wigwag frequency were calculated using Eqs. (7) and (8), respectively,

7 $$E(x) = {\mathrm{exp}}\left( {\mu + \frac{{\sigma ^2}}{2}} \right)$$

8 $$V(x) = \left( {e^{\sigma ^2} - 1} \right)e^{2\mu + \sigma ^2}$$

where *μ* is the mean, *σ* is the standard distribution.

An assigned value of 3 was used as a coefficient to approximate the angle of wigwag self-motions of the head. If the input difference was acceptable $\left( {\left| {R\left( {\theta _t} \right) - L\left( {\theta _t} \right)} \right| \le {\mathrm{\tau }}} \right)$, the body angle did not change using Eq. (9):

9 $$\varphi _{t \,+\, k} = \varphi _t$$

Wigwag self-motion was considered to add a random parameter $\xi$, which is a randomly generated parameter with a distribution that fits the normal distribution^29^ (Fig. 6b, c) using Eqs. (10)–(12):

10 $$\begin{array}{ccccc}\\ \varphi _{t \,+\, k} = & \varphi _t - 3\left( {\left( {R\left( {\theta _t} \right) - L\left( {\theta _t} \right)} \right) - {\mathrm{\tau }}} \right) + \xi ,\\ \\ & \left( {\left| {R\left( {\theta _t} \right) - L\left( {\theta _t} \right)} \right| \,> \, {\mathrm{\tau }} \cap R\left( {\theta _t} \right) \,< \,L\left( {\theta _t} \right)} \right)\\ \end{array}$$

11 $$\begin{array}{ccccc}\\ \varphi _{t \,+\, k} = & \varphi _t + 3\left( {\left( {R\left( {\theta _t} \right) - L\left( {\theta _t} \right)} \right) - {\mathrm{\tau }}} \right) + \xi ,\\ \\ & \left( {\left| {R\left( {\theta _t} \right) - L\left( {\theta _t} \right)} \right|\, > \,{\mathrm{\tau }} \cap R\left( {\theta _t} \right) \,> \,L\left( {\theta _t} \right)} \right)\\ \end{array}$$

12 $$\varphi _{t \,+\, k} = \varphi _t + \xi ,\left( {\left| {R\left( {\theta _t} \right) - L\left( {\theta _t} \right)} \right| \le {\mathrm{\tau }}} \right)$$

The coordinates of the predicted planarian position at time *t* were calculated using Eq. (13):

13 $$\left( {x_{t \,+\, k},y_{t \,+\, k}} \right) = \left( {x_t + \sin \left( {\varphi _t} \right),y_t + \cos \left( {\varphi _t} \right)} \right)$$

### Histology

For whole-mount immunohistochemistry, planarians were stained using the following dilutions of antibodies: 1/2000 rabbit anti-planarian arrestin^16^, 1:2000 rabbit anti-planarian GAD^28^, 1:2000 mouse anti-planarian TPH^31^. The plasmids pBluescript SK (−) containing planarian *chat*^59^, *gad*^28^*, GABAA-RBa*, *GABAB-Ra, opsin*^6^, *snap25*^24^, *syt*^60^, *tbh*^61^, and *th*^62^ cDNAs were used as templates for synthesizing digoxigenin (DIG)-labeled antisense RNA probes (Roche Diagnostics). Planarians were treated with 1% HNO_3_, 50 mM MgCl_2_ solution for 5 min at room temperature and fixed in 4% paraformaldehyde, 5% methanol, 50% PBS solution for 30 min at room temperture. Fixed animals were subjected to in situ hybridization with appropriate probes. For visualization of fluorescent color a TSA kit (Thermo Fisher Scientific) was used according to the manufacturer’s instructions. Cell nuclei were labeled with Hoechst 33342. Fluorescence was detected with a confocal laser scanning microscope (FV10i, Olympus).

### RNA interference

Double-stranded RNAs (dsRNAs) were synthesized from appropriate cDNA clones. dsRNA was injected into the posterior intestinal duct of planarians for three successive days using an injector (Drummond Scientific). Four hours after the injection, animals were amputated posterior to the auricles, and the resulting regenerants were used in the analysis at 7 days of regeneration. Control animals were injected with dsRNA for green fluorescent protein, a gene that is not found in planarians.

### Reverse transcription and quantitative PCR analysis

Animals were divided into the head and body. The eye-rich fragments were collected surgically using a capillary glass pipette. Total RNA was extracted from planarians using ISOGEN-LS (Nippon Gene), and first-strand cDNA was synthesized from 1 µg total RNA using a QuantiTect Reverse Transcription Kit (Qiagen). Quantitative analysis of the amount of each gene product was performed using the real-time polymerase chain reaction (PCR) machine (7900HT, Thermo Fisher Scientific). Each reaction (10 µl) contained 1× QuantiTect SYBR Green PCR Master Mix (Qiagen), gene-specific primer at 0.3 µM, and 1 µl diluted (1:20) cDNA solution as template. At least four technical replicas and at least three biological replicas were done. The PCR primers used are listed in Table 1. Expression levels were normalized by *GAPDH* gene expression.

**Table 1:** Oligonucleotide primer sequences used for qRT-PCR assays

Names	Sequences
*gad* forward	5′-AAAAGTCATCGCTATTTACTGAATGGAA-3′
*gad* reverse	5′-CACAACTTGAACACATCATTTCGTCTAC-3′
*GABAA-RBa* forward	5′-TTTCGTCTTCCAAACTTATTTACCATCA-3′
*GABAA-RBa* reverse	5′-AGAACACAAATACAAAACAAACCACGAG-3′
*GABAB-Ra* forward	5′-TTATAATTGGTTGGTATCCTGATGATT-3′
*GABAB-Ra* reverse	5′-GGAATTTTATCTTCTGTGTTTCTCCATT-3′
*opsin* forward	5′-AGCAACAAAACCAAGTAAATACCAAAGT-3′
*opsin* reverse	5′-ACATTGAAACAAACATTTTTACGCCTAT-3′
*snap25* forward	5′-AGGCTGGTATCTCTACACTTGTTATGCT-3′
*snap25* reverse	5′-ATATTTTGATCCATTTCATCTTCTCTGG-3′
*GAPDH* forward	5′-ACCACCAACTGTTTAGCTCCCTTAG-3′
*GAPDH* reverse	5′-GATGGTCCATCAACAGTCTTTTGC-3′

## Electronic supplementary material

Supplementary file

## Data Availability

The sequences of *GABAA-RBa* and *GABAB-Ra* reported here have been deposited in the DNA Data Bank of Japan (DDBJ) and National Center for Biotechnology Information (NCBI) (Accession numbers LC035387 and LC035388). All materials except for some primary antibodies used in the present work described in the manuscript are available from standard commercial sources (Thermo Fisher Scientific, Sigma, Roche, Takara Bio) or from the corresponding author (T.I.). The anti-planarian arrestin and anti-planarian tryptophan hydroxylase used are an antiserum and culture supernatant, respectively, and therefore the distribution of these antibodies might become restricted.
