# Supplementary material for: Coordination between binocular field and spontaneous self-motion specifies the efficiency of planarians’ photo-response orientation behavior
Source: Commun Biol. 2018 Sep 21;1:148. doi: 10.1038/s42003-018-0151-2 (PMC6155068; doi:10.1038/s42003-018-0151-2)
Supplement: Supplementary file 1 — Supplementary file [file 42003_2018_151_MOESM1_ESM.pdf]

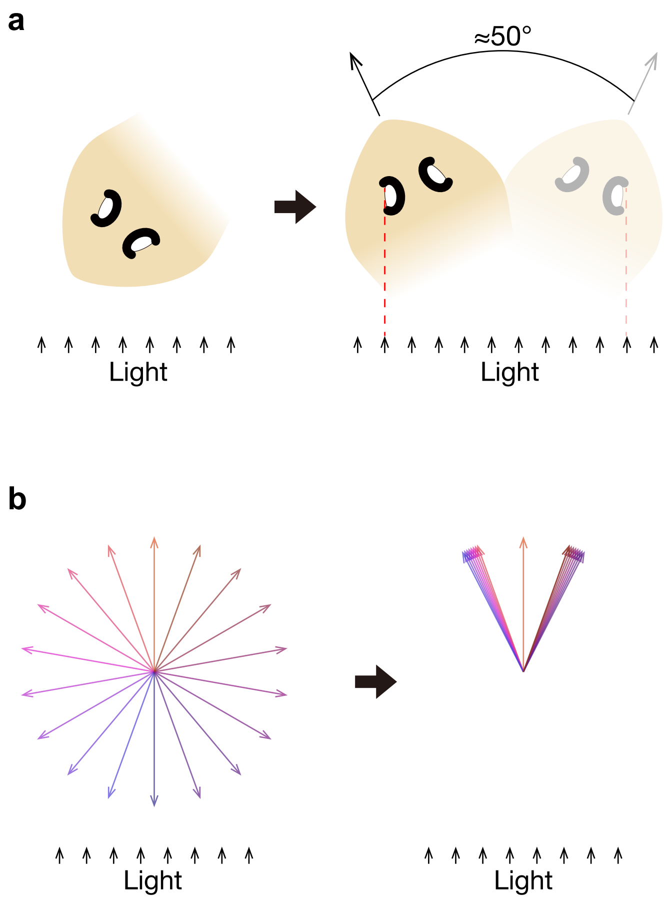

Supplementary Figure 1. Schematic drawings of principle of moving in the twin-tail paths.
(**a**) A planarian facing toward the light source turns its body and orients in a direction that no longer receives the light. The orientation is oblique to the direction of the light due to the obliqueness of the visual field. (**b**) Planarians oriented in a random direction change their orientation, and consequently the orientation falls into two directions.

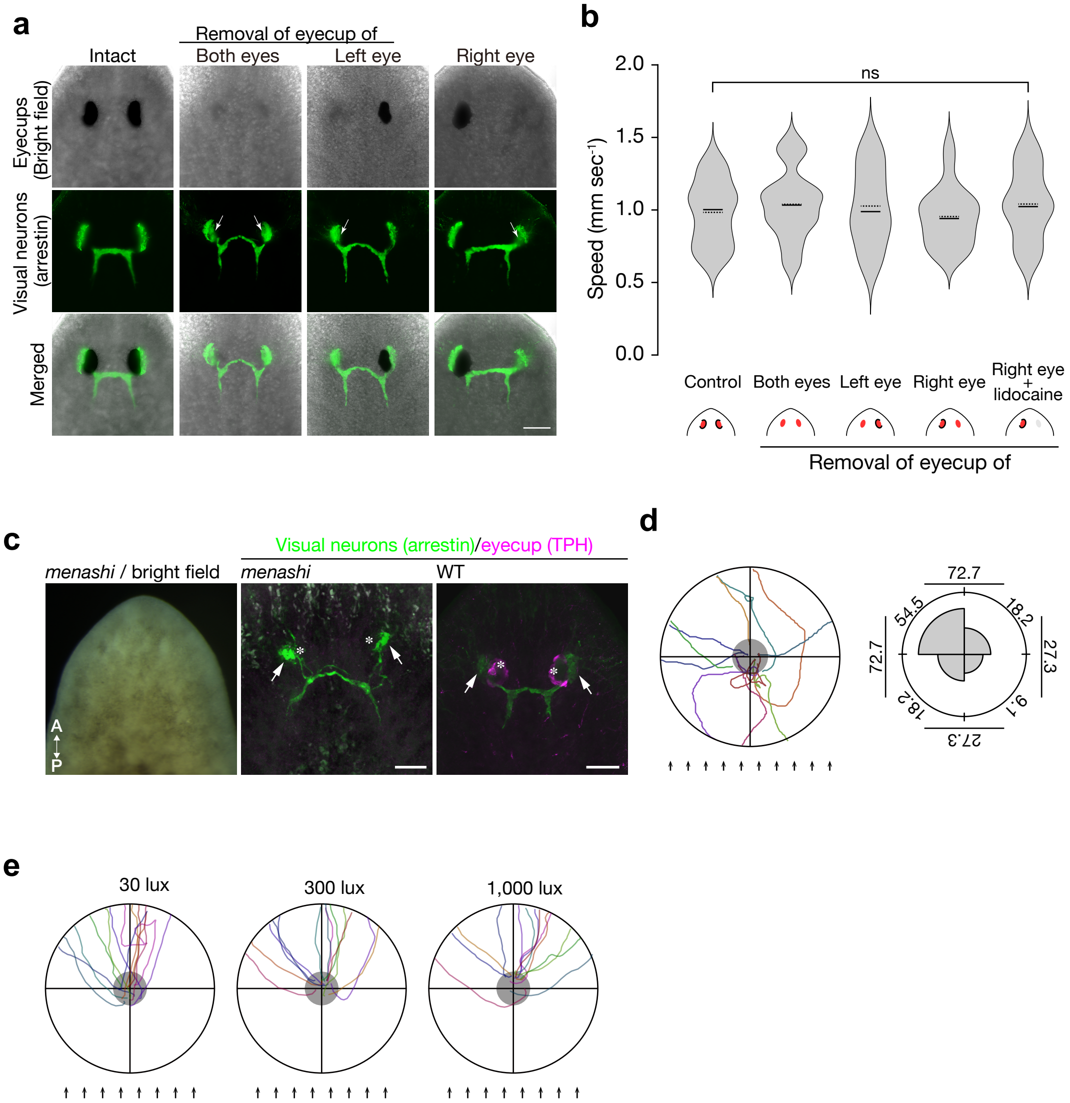

Supplementary Figure 2. Visual neurons and locomotor activity after removal of eyecups.
(**a**) Visual neurons (green) visualized by immunostaining of arrestin of *D. japonica* after pigment eyecup removal. Pigment eyecups were removed, but the visual neurons were normal. Arrows indicate rhabdomeric dendrites. Scale bar, 100 µm. (**b**) Distribution of the speed of individuals during photo-response orientation behavior of eyecup-removed *D. japonica* shown in violin plots. Horizontal solid lines indicate median value, and horizontal dashed lines indicate average value in violin plots. ns, not significantly different. (**c**) Visual neurons (green) visualized immunostaining of arrestin and pigment cups (magenta) visualized by immunostaining of TPH of the *menashi* mutant. Asterisks, pigment cups; Arrows, visual neurons. Scale bar, 100 µm. (**d**) Trajectories and rose plot in OA1L of the *menashi* mutant. Arrows, light source. (**e**) Trajectories in OA1L with exposure to 30, 300, or 1,000 lux intensity of light.

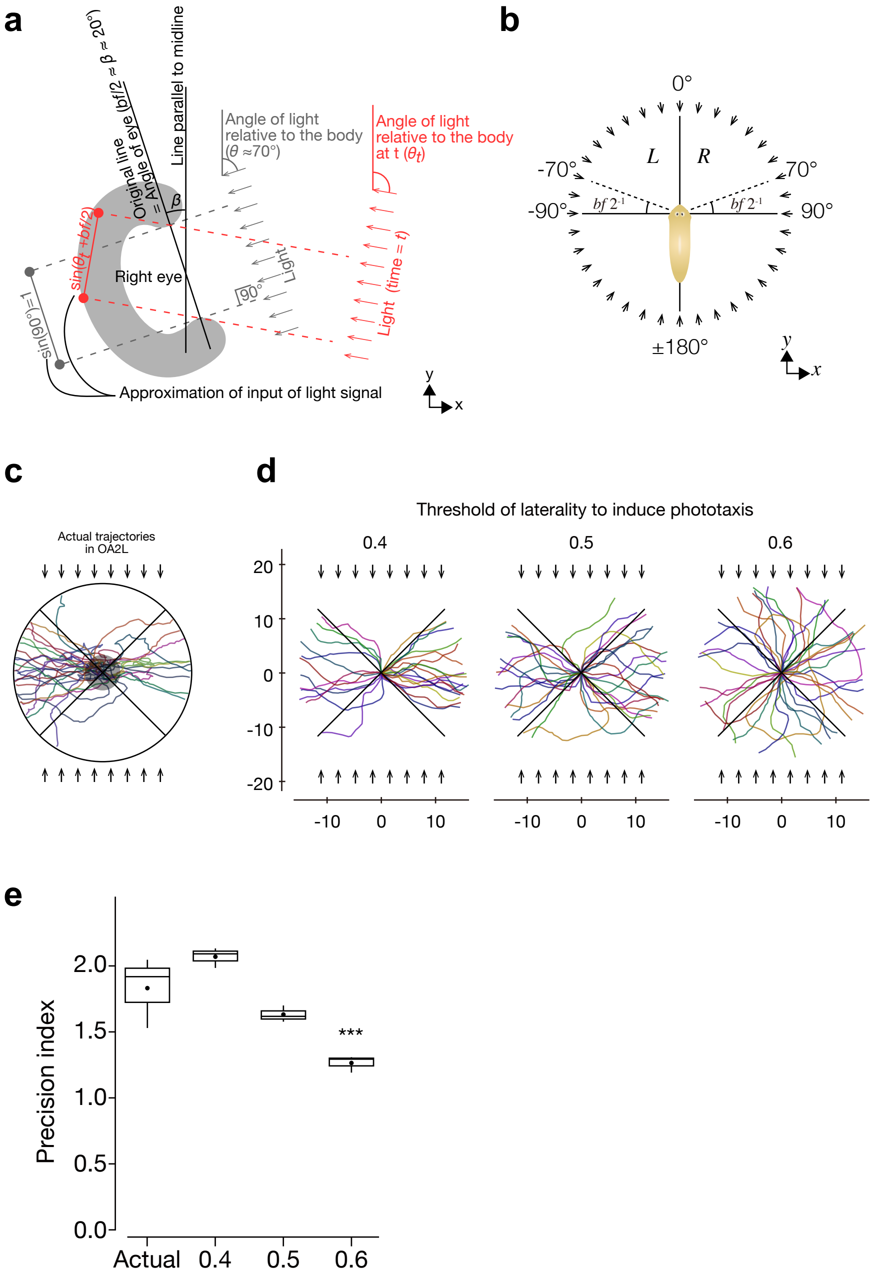

Supplementary Figure 3. Approximation of input of light and response threshold of the body reaction.
(**a**) The input light intensity in each eye was approximated by the area of the surface receiving light represented by a sine function due to the semicircular shape of the pigment cup, and was assessed by the angle of the light direction. This drawing represents the right eye. Due to the obliqueness of the eyes of approximately 20° ($\beta \approx bf\,2^{-1}$) relative to the anterior-posterior body axis if the light source is located at 70° (light source indicated in gray), the intensity of light is maximal, and is defined as 1.0. θt is the light direction relative to the animal body axis t (light source indicated in red). (**b**) Schematic drawing of the light angle relative to the body. Arrows, light sources. (**c**) Actual trajectories in the orientation assay with two light sources (OA2L). Planarians escaped in paths perpendicular to both of the light sources when they were illuminated from two opposite directions. Arrows indicate rays of light. n = 34. (**d**) Simulated trajectories in OA2L with a response threshold parameter of 0.4, 0.5 or 0.6. At a response threshold of 0.5, the simulated trajectories were similar to the actual trajectories in c. At a response threshold of 0.6, trajectories were random. (**e**) Precision index (the inverse of the circular standard deviation calculated from the orientation of movement) of the orientation of movements. ***, $p < 0.005$.

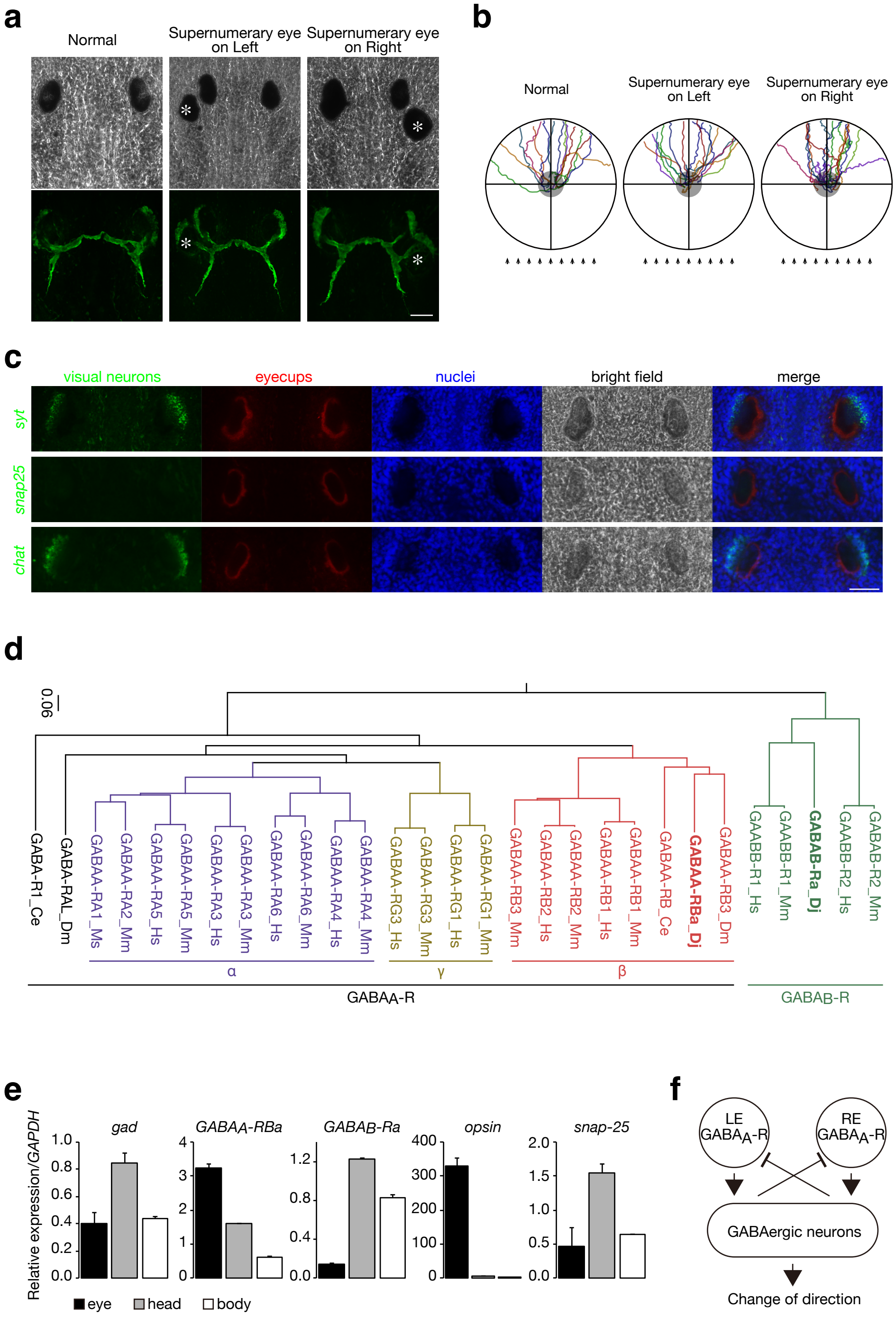

Supplementary Figure 4. Neural pathway in the brain for photo-recognition.
(**a**) *D. japonica* with a supernumerary eye visualized by immunohistochemistry with an anti-arrestin antibody (green). Bar, 50 µm. *, supernumerary eye. (**b**) OA1L of *D. japonica* with a supernumerary eye. *D. japonica* possessing a supernumerary eye showed normal photo-response orientation behavior, even though the signal input was expected to be stronger on one side, indicating that the neural network in the brain compensates for the laterality of the signal input to achieve light-evasion behavior. Arrows, light source. (**c**) Expression patterns of the planarian *syt*, *snap25*, and *chat* genes in visual neurons indicated by green. The *syt* and *chat* genes are expressed in visual neurons, but the *snap25* gene is not expressed in visual neurons. Eyecups were stained with anti-TPH antibody, indicated by red. Nuclei were stained with Hoechst33342, indicated by blue. Scale bar, 20 µm. (**d**) Phylogenetic tree of representative GABA receptors obtained by aligning GABA receptors from various species. Ce, *C. elegans*; Dm, *D. melanogaster*; Dj, *D. japonica*; Hs, *H. sapiens*; Mm, *M. musculus*. The scale bar indicates substitutions per site. (**e**) Relative gene expression levels of GABA-related genes as assessed by qRT-PCR. Expression levels were normalized by those of intact animals. A high expression level of the *GABAA-RBa* gene was detected in the eye-rich fraction, whereas the expression levels of the gad and *GABAB-Ra* genes were lower in the eye-rich fraction than in the head. The opsin gene (specifically expressed in visual neurons) and *snap25* gene (not expressed in visual neurons) were used as positive and negative controls, respectively. men ± SEM. (**f**) Schematic illustration of a potential inhibitory neural pathways regulating laterality to induce body reactions in accordance with the all-or-none theory in planarian.

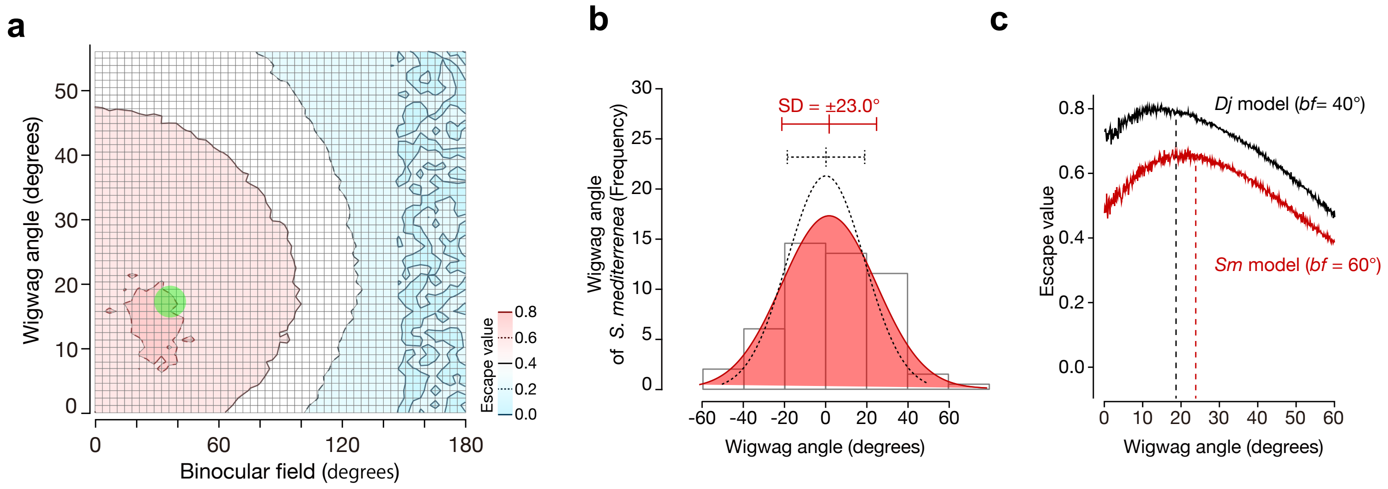

Supplementary Figure 5 Escape value with a correlation between the angle of the binocular field and the angle of the wigwag self-motion.
(**a**) Escape values were plotted against two variables: the wigwag angle and binocular field. Wigwag angle indicates the value of standard deviation. The contour lines indicate the value of escape value. The optimal angle of the binocular field is approximately 20°- 40° and the optimal wigwag angle is approximately 10°-20°. Planarians with eyes with a binocular field less than 40 degrees or a wigwag angle less than 10° showed a low escape value. The green circle indicates the actual value of *D. japonica*. The simulated optimal combination between the wigwag angle and the binocular field accords with the actual value of *D. japonica*. n = 1,000. (**b**) Density plot showing distribution of wigwag angle of *S. mediterranea* indicated in red. Dashed line indicates wigwag angle of *D. japonica*. The angle of head-swaying of *S. mediterranea* was wider than that of *D. japonica*. (**c**) Escape value is plotted against wigwag standard deviation, simulated with the two-parameter set of *D. japonica* model (*bf* ≈ 40) indicated in black and S. mediterranea model (*bf* ≈ 60) indicated in red. Dashed vertical lines indicate actual values of each planarian. The optimal value of the *Sm* model possessing a wider binocular field is shifted to larger wigwag angles compared to the *Dj* model possessing a smaller binocular field.
